# Increased Early Processing of Task-Irrelevant Auditory Stimuli in Older Adults

**DOI:** 10.1371/journal.pone.0165645

**Published:** 2016-11-02

**Authors:** Erich S. Tusch, Brittany R. Alperin, Phillip J. Holcomb, Kirk R. Daffner

**Affiliations:** 1 Center for Brain/Mind Medicine, Division of Cognitive and Behavioral Neurology, Department of Neurology, Brigham and Women's Hospital, Harvard Medical School, 221 Longwood Avenue, Boston, MA, 02115, United States of America; 2 Department of Psychology, Oregon Health and Science University, 3181 S.W. Sam Jackson Park Rd., Portland, OR, 97239, United States of America; 3 Department of Psychology, Tufts University, 490 Boston Avenue, Medford, MA, 02155, United States of America; Center for BrainHealth, University of Texas at Dallas, UNITED STATES

## Abstract

The inhibitory deficit hypothesis of cognitive aging posits that older adults’ inability to adequately suppress processing of irrelevant information is a major source of cognitive decline. Prior research has demonstrated that in response to task-irrelevant auditory stimuli there is an age-associated increase in the amplitude of the N1 wave, an ERP marker of early perceptual processing. Here, we tested predictions derived from the inhibitory deficit hypothesis that the age-related increase in N1 would be 1) observed under an auditory-ignore, but not auditory-attend condition, 2) attenuated in individuals with high executive capacity (EC), and 3) augmented by increasing cognitive load of the primary visual task. ERPs were measured in 114 well-matched young, middle-aged, young-old, and old-old adults, designated as having high or average EC based on neuropsychological testing. Under the auditory-ignore (visual-attend) task, participants ignored auditory stimuli and responded to rare target letters under low and high load. Under the auditory-attend task, participants ignored visual stimuli and responded to rare target tones. Results confirmed an age-associated increase in N1 amplitude to auditory stimuli under the auditory-ignore but not auditory-attend task. Contrary to predictions, EC did not modulate the N1 response. The load effect was the opposite of expectation: the N1 to task-irrelevant auditory events was smaller under high load. Finally, older adults did not simply fail to suppress the N1 to auditory stimuli in the task-irrelevant modality; they generated a larger response than to identical stimuli in the task-relevant modality. In summary, several of the study’s findings do not fit the inhibitory-deficit hypothesis of cognitive aging, which may need to be refined or supplemented by alternative accounts.

## Introduction

The ability to focus on task-pertinent information and limit interference from task-irrelevant stimuli is critical to the execution of goal-directed behaviors [1, 2]. The inhibitory deficit hypothesis of cognitive aging proposes that older adults’ failure to adequately inhibit the processing of task-irrelevant information clutters and disrupts capacity-limited information processing and is a major source of age-related decline in cognitive performance [3–5]. There is strong evidence that older adults are less adept at filtering irrelevant stimuli [6–9]. Older individuals tend to exhibit larger behavioral costs in response to distracters on Stroop [10–12], Simon [13, 14], reading-with-distraction [15, 16], and listening-in-noise [17, 18] tasks. Moreover, older adults demonstrate reduced ability to suppress neural activity in response to task-irrelevant stimuli in studies employing functional magnetic resonance imaging (fMRI) and event-related potentials (ERPs) [2, 6, 8, 9, 19–22].

Selective attention has been conceptualized as reflecting the activity of executive control functions over sensory input [23, 24]. Lavie and colleagues [6, 25, 26] have argued that the executive component of working memory allows individuals to actively maintain current processing priorities online that differentiate between relevant targets and irrelevant distracters [27–29]. Higher cognitive load of the primary task results in increased interference by irrelevant distracters because, in this context, executive control functions are less available to actively maintain stimulus-processing priorities [26].

An important study testing the inhibitory deficit hypothesis was conducted by Alain and Woods [2], who used ERPs in a cross-modal paradigm to determine if there were age-related increases in the neural processing of task-irrelevant auditory stimuli. Participants were instructed to ignore auditory events while they performed a visual discrimination go/no go task. The auditory N1, a frontocentral negativity peaking 50–150 ms after stimulus onset [30], was employed as an index of early perceptual processing of auditory stimuli. The N1 has been shown to be modulated by bottom-up factors. For instance, stimuli that have greater perceptual salience (e.g., due to being louder, or more abrupt in onset) elicit a larger N1 [30, 31]. The N1 is also influenced by top-down control factors. For example, during dichotic listening tasks, the N1 amplitude is greater in response to auditory stimuli presented to the attended than the ignored ear [32]. In their study of aging, Alain and Woods found a robust age-related increase in the N1 amplitude in response to task-irrelevant auditory stimuli that participants were instructed to ignore, which the investigators interpreted as providing strong evidence in support of the inhibitory deficit hypothesis of aging. Other studies have found similar age-related changes in early processing of ignored auditory stimuli, further supporting this hypothesis [33, 34].

Several limitations of the seminal paper by Alain and Woods [2] are representative of limitations commonly found in the literature investigating neural markers of age-related decline in inhibition [6, 8, 9, 20, 21]. First, Alain and Woods [2] did not include an auditory attend condition, leaving open the possibility that the observed age-related increase in the amplitude of the N1 wave was not specific to a condition in which auditory stimuli were intended to be ignored. Second, the Alain and Woods study did not explicitly match different age groups in terms of cognitive capacity or performance on the experimental task, making it difficult to interpret whether differences in neural activity between groups were due to age or other factors, such as executive capacity (EC), perceived task difficulty, or task performance [22, 35]. Consistent with proposed mechanisms underlying inhibitory activity, one would expect the EC of participants to modulate performance on tasks that require the inhibition of processing task-irrelevant information [36, 37]. Similarly, one would anticipate the level of cognitive difficulty of the primary visual task to influence the amount of remaining capacity-limited resources available to filter task-irrelevant events [26]. According to this framework, research on age-associated changes in neural activity underlying selective attention needs to account for the potential impact of group differences in EC and level of task difficulty. Finding that differences across age groups in the processing of task-irrelevant auditory stimuli persist after matching them in terms of EC and primary visual task load would greatly strengthen the claim of a decline in inhibitory processing specific to aging itself.

Third, the Alain and Woods investigation included a relatively small number of participants (n = 38) in three groups, young adult (mean age 23.6 years old), middle-aged (mean age 43.3 years old), and young-old (mean age 65.7 years old), leaving uncertain whether the pattern of findings would continue in old-old age or be replicated with a larger sample. Fourth, although hearing loss was measured, the study did not control for age-related differences in auditory acuity, which may have impacted their findings. A final limitation of the Alain and Woods study is that the N1 wave was measured on averaged waveforms alone. To draw strong inferences about age-related changes in the operations indexed by the N1, one needs to demonstrate that the differences observed in the grand average waveforms are not due to changes in components that temporally or spatially overlap with the N1 (e.g., the anterior P1 that precedes it or the anterior P2 that follows it), which may also be influenced by age [38].

The current investigation attempts to address each of these limitations. First, it included conditions comprised of identical kinds of physical stimuli, differing only in the instructions given [29] such that participants focused on either a visual or auditory task with cross-modal distracters. Second, it aimed to match age groups in terms of EC and accuracy on the primary visual task. EC was measured by performance on neuropsychological tests [39, 40]. Both a low and high task load condition of the visual (auditory-ignore) task were included. The low load condition was the same for all participants (i.e., one visual target stimulus). For the high load condition, the number of visual targets was determined individually for each participant by a titration process that aimed to keep task performance consistent across participants and age groups. This allowed us to draw inferences about age- rather than performance-related differences in neural activity. Third, the current study comprised a much larger sample of participants (n = 114) and included an old-old age group (80 years and older). Fourth, the volume of the auditory stimuli was adjusted based on formal audiologic examination to compensate for participants' individual hearing loss, following methods used by Fabiani & Friedman [41]. Finally, to address the issue of potentially overlapping components influencing findings regarding the N1 wave, ERP data were subjected to temporospatial principal component analysis (PCA) to determine if the results were consistent with those found by analyzing average waveforms (see Supporting Information for this analysis) [42].

The inhibitory deficit theory of cognitive aging leads to the following set of hypotheses: 1) There will be an age-associated increase in the N1 to task-irrelevant auditory stimuli under the auditory-ignore task, but not the auditory-attend task. If age differences are found in the auditory-attend task, one would expect the magnitude of these effects to be smaller than those observed under the auditory-ignore task. The anticipated age-related increase of early processing should continue into old-old age, a group often not included in previous studies. Additionally, this age-related increase of early processing will be independent of the influence of age-related hearing loss. 2) EC will modulate age-related increases in auditory N1 amplitude during the auditory-ignore task. Older individuals with higher EC will be more proficient at inhibiting neural responses to task-irrelevant auditory stimuli than their counterparts with lower EC, leading to an attenuation of age-related increases in the N1 to task-irrelevant auditory stimuli. 3) Working memory load in the primary visual (auditory-ignore) task will modulate age-related increases in the N1 to auditory stimuli. Higher cognitive load during the primary visual task should lead to reduced executive control resources available to actively maintain stimulus processing priorities, resulting in increased processing of task-irrelevant auditory events. Given older adults’ hypothesized vulnerability to interference, one would predict the magnitude of age-related increases in early processing of auditory stimuli during the auditory-ignore task to be larger under the high load condition.

The inhibitory deficit hypothesis does not directly address the potential impact of stimulus salience on age-related changes in inhibition. Therefore, an explicit prediction regarding this issue was not generated. However, because salient stimuli tend to be more challenging to ignore than non-salient, repetitive stimuli [43], one might expect that older adults would have particular difficulty inhibiting their processing. In the current study, this issue was explored by including an analysis of both repetitive standard and infrequent novel auditory stimuli; rare auditory stimuli were not included. In the auditory-attend task, rare stimuli were designated as targets. Target stimuli tend to be processed very differently from non-target stimuli [44]. Including rare target auditory stimuli in the analysis would have led to a confound between direction of a participant’s attention (toward stimuli in the auditory vs. the visual modality) and the distinction between targets and non-targets, making the results difficult to interpret.

## Methods

### Participants

Participants were recruited through community announcements in the Boston metropolitan area, including the Harvard Cooperative Study on Aging. The study was approved by the Partners Human Research Committee (protocol number 2008p001897). All participants completed written informed consent. Participants also completed a detailed screening evaluation that included a structured interview to obtain a medical, neurological, and psychiatric history; a formal neurological examination, audiologic evaluation, and test of visual acuity via Snellen Wall chart; and the completion of a neuropsychological test battery and questionnaires surveying mood and daily living activities.

To be included in this study, participants had to be English-speaking, have ≥ 12 years of education, have a Mini Mental State Exam (MMSE) [45] score ≥ 26, and have an estimated intelligence quotient (IQ) on the American National Adult Reading Test (AMNART) [46] ≥ 100. Participants were divided into four age groups: 18 to 32 years (young), 40 to 59 years (middle-aged), 60 to 79 years (young-old), and ≥ 80 years old (old-old). Participants were excluded if they had a history of CNS diseases or major ongoing psychiatric disorders based on DSM-IV criteria [47], focal abnormalities on neurological examination consistent with a CNS lesion, a history of clinically significant medical diseases, or corrected visual acuity worse than 20/80. Participants were also tested with pure tone audiometry in which hearing thresholds were tested at 250, 500, 1000, 2000, and 4000 Hz, and excluded if they demonstrated the following abnormalities: > 40 dB mean loss across frequencies, > 20 dB difference between ears at any frequency, or > 30 dB difference between the best and worst threshold [48]. Participants were excluded if their mean percentile performance relative to age-appropriate norms across selected neuropsychological tests (described below) was in the bottom third (below the 33^rd^ percentile) in order to exclude older individuals who may be suffering from mild cognitive impairment or the very early stages of a dementing illness. Participants were paid for their time.

To avoid conflating changes in neural activity that are specifically due to differences in age with those due to differences in cognitive ability or task performance, it is crucial to limit differences between age groups in these factors [49–51]. Daselaar and Cabeza [50] argue in favor of grouping participants based on a battery of neuropsychological tasks that are standardized and therefore generalizable. Due to the role of top-down control in inhibition of early stimulus processing, as well as the role of executive capacity in normal cognitive aging [9, 26, 52], age groups were matched in terms of EC relative to age-appropriate norms on neuropsychological tests.

In measuring EC, we followed the suggestion of many investigators who emphasize processes that include working memory, initiation, monitoring, and inhibition, and advocate the use of several neuropsychological tests to assess this complex group of functions [53–55]. Tests of EC were selected that had well-established norms across a wide range of ages. Tests included: 1) Digit Span Backward subtest of the Wechsler Adult Intelligence Scale-IV (WAIS-IV) [56], which measures maintenance and manipulation operations of working memory; 2) Controlled Oral Word Association Test (COWAT) [57], which indexes initiation, self-generation, and monitoring; 3) Trail-Making Test Parts A and B [58], which measures planning/sequencing, set shifting, and inhibition; 4) WAIS-IV Digit-Symbol Coding, which assesses sustained attention, cognitive speed, and inhibition; and 5) WAIS-III or WAIS-IV Letter-Number Sequencing, which assesses monitoring, inhibition, and manipulation. Note that WAIS III Letter-Number Sequencing was administered for young-old and old-old participants and WAIS IV Letter-Number Sequencing was administered for young and middle-aged participants in order to take advantage of the wider range of age-appropriate norms in the former version of the test. Participants were divided into two EC groups: high EC participants were those whose mean percentile score based on age-appropriate norms was > 66.6; average EC participants had a mean percentile score based on age-appropriate norms of 33.3–66.6. For participants older than 85, the closest possible age-appropriate norms were used.

### Experimental Procedure

**Fig 1:**
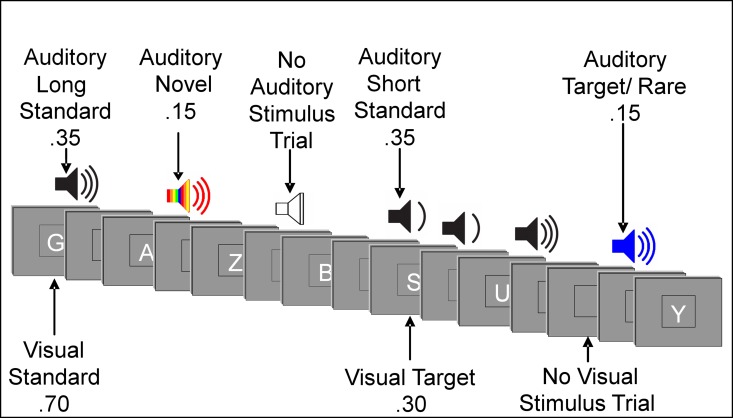
Illustration of an Experimental Run. Example sequence of auditory and visual stimuli. Participants performed an oddball task in each modality while instructed to ignore the other. Targets in the auditory-attend task were designated by frequency, and targets in the visual task were specific letters.

The experiment consisted of forced-choice oddball paradigms under an auditory-attend or visual (auditory-ignore) task. Stimuli were presented using E-Prime software [59]. Participants were instructed to respond to target stimuli and non-target stimuli with opposite mouse clicks (e.g., left click for target stimuli and right click for non-target stimuli). The hand used for the target response was counterbalanced across participants. During the auditory-attend task, participants were instructed to respond to sounds identified by frequency and ignore letters visually presented on a computer monitor. During the auditory-ignore (visual) tasks, participants were instructed to respond to letters and ignore sounds. Order of stimulus presentations varied randomly across blocks within tasks and across tasks. Presentation of letters and sounds did not temporally overlap (Fig 1).

Visual stimuli appeared one at a time within a fixation box that remained on the screen at all times and subtended a visual angle of ~3.5° x 3.5° at the center of a high-resolution computer monitor. Visual stimuli subtended an angle of 2.5° along their longest dimension and were presented for 200 ms. Target letters comprised 30% of visual stimuli. Non-target letters comprised 70% of visual stimuli. Auditory stimuli were presented one at a time with a minimum intensity of 75dB SPL. Decibel level was adjusted for any participant for whom pure tone audiometry showed a mean hearing loss (across tested frequencies) of 0–40 dBs by increasing (from 75dB) the intensity of sounds by the mean decibel hearing loss [48]. Standard auditory stimuli, comprising 70% of auditory stimuli, were 250 Hz pure tones presented for a duration of either 250 ms (35%) or 125 ms (35%). Rare auditory stimuli, comprising 15% of auditory stimuli, were 500 Hz pure tones of either long (250ms) or short (125ms) duration and served as the designated targets in the auditory-attend task. Targets were designated by frequency; participants were instructed to identify and respond to sounds based on frequency, regardless of duration. Short and long rare stimuli were not presented in equal proportion: each comprised 80% or 20% of total rare auditory stimuli, counterbalanced across participants. Novel auditory stimuli were complex, environmentally derived or synthesized sounds presented for a duration of 250 ms, comprising 15% of auditory stimuli. Each novel auditory stimulus was unique within and between tasks.

Auditory stimuli had ~20 ms rise/fall times. The inter-stimulus interval (ISI) between auditory and visual stimuli varied randomly between 315–665 ms (mean ~490 ms) with 1 ms steps and a rectangular distribution. In addition to the 1600 visual (800) and auditory (800) stimulus trials, there were 200 auditory and 200 visual trials devoid of a stimulus. For visual stimuli, a no-stimulus trial appeared as a blank presentation box. For auditory stimuli, a no-stimulus trial was a period of silence when an auditory stimulus was to be expected.

The tasks included 1600 stimulus trials divided into 16 blocks. The auditory and visual tasks were both comprised of eight blocks each. In the visual task, 30% of trials presented visual stimuli (letters) from the target category and 70% of trials presented stimuli from the standard category. Under low task load, one letter was designated as a target. Under the high task load, the number of unique target letters in the target category varied across participants and was determined by an individual’s performance on a titration task. During the titration task, participants were tested on consecutive blocks of the visual task without auditory stimuli. The number of unique letters designated as target stimuli varied across blocks. The number of target letters for which participants scored closest to 80% accuracy (target hit ratio—false alarm ratio) was chosen to be used for the high visual task load condition. This procedure was adopted to help ensure that the level of difficulty of the primary visual task was similar across participants from different age groups. Although the number of visual target letters varied across participants from three to nine, the percentage of trials categorized as target events was the same for everyone.

During the auditory-attend task, primary task load of the forced-choice auditory oddball task did not vary. Participants were instructed to respond to rare auditory stimuli designated by frequency as target tones by clicking one side of the mouse, and to respond to all other auditory stimuli by clicking the other side of the mouse; visual stimuli were to be ignored. For one four-block section, one unique visual letter was presented on 30% of trials, matching the number used for the low load condition of the visual-attend (auditory-ignore) task. For the other four-block section, the number of unique letters presented on 30% of trials matched that used for the high load condition of the visual attend task. The other letters were randomly selected for the remaining 70% of the trials. Thus, unique letters appeared with the same probabilities across blocks of the visual-attend (auditory-ignore) and auditory-attend tasks. This procedure was adopted to ensure that participants were exposed to physically identical kinds of stimuli across all tasks.

Participants visited the laboratory on three occasions. During the first visit, neuropsychological testing, audiometry, and the visual task load titration procedure were completed. During the remaining two visits, the auditory and visual tasks, with concurrent ERP recordings, were completed. The latter two visits were scheduled approximately two weeks apart from each other to reduce any potential order effects. Each task took approximately 45 minutes to complete. One task was completed per visit, and task order was counterbalanced across participants. In the current paper, only ERP data on novel and standard long-duration auditory stimuli will be presented.

### ERP Recordings

An ActiveTwo electrode cap (Behavioral Brain Sciences Center, Birmingham, UK) was used to hold to the scalp a full array of 128 Ag-AgCl BioSemi (Amsterdam, The Netherlands) “active” electrodes whose locations were based on a pre-configured montage. Electrodes were arranged in equidistant concentric circles from 10–20 system position Cz. In addition to the 128 electrodes on the scalp, 6 mini bio-potential electrodes were placed over the left and right mastoid, beneath each eye, and next to the outer canthi of the eyes to check for eye blinks and vertical and horizontal eye movements. EEG activity was digitized at a sampling rate of 512 Hz and filtered offline with a bandwidth of .016–100 Hz.

### Data Analysis

Statistical analyses were conducted using Statistical Package for Social Sciences (SPSS) version 23. Significance was set at *p* < .05. Demographic variables and overall percentile performance on the neuropsychological tests for the groups were compared using one-way analysis of variance (ANOVA). Mean target accuracy and mean reaction time (RT) during the visual-attend (auditory-ignore) condition were measured. E-Prime software was used to generate the behavioral data. A correct response was considered a hit if it occurred between 200–1000 ms after stimulus presentation. Target stimuli correctly responded to (target hits) and stimuli incorrectly identified as targets (false alarms) were measured in order to determine an overall accuracy score (percent target hits minus percent false alarms). Due to technical issues during data collection, behavioral data recorded by E-Prime are missing for two young participants. Their ERP data could still be analyzed because responses (correct/incorrect) were coded in the BioSemi EEG files.

EEG data were analyzed using ERPLAB [60] and EEGLAB [61] toolboxes that operate within the MATLAB framework. Raw EEG data were resampled to 256 Hz and referenced off-line to the algebraic average of the right and left mastoids. EEG signals were filtered using an IIR bandpass filter with a bandwidth of .03–40 Hz for young and middle-aged participants and .03–30 Hz for young-old and old-old participants (12 dB/octave roll-off for all). Eye artifacts were removed through an independent component analysis. Individual channels that revealed, upon visual inspection, a consistently different pattern of activity from surrounding channels were corrected with the EEGLAB interpolation function. EEG epochs for the two stimulus types (standard and novel auditory stimuli) across two attention conditions (auditory-attend and auditory-ignore task) and two task loads (low and high) were averaged separately. The sampling epoch for each trial lasted for 1200 ms, including a 200 ms pre-stimulus period that was used to baseline correct the ERP epochs. Trials were discarded from the analyses if they contained baseline drift or movement artifacts greater than 90 μV. Only trials with correct responses were included in the analyses. Participants were excluded from further analyses if their data were excessively noisy due to frequent contamination by motion artifacts or alpha waves.

### Average Waveform Analysis

**Fig 2:**
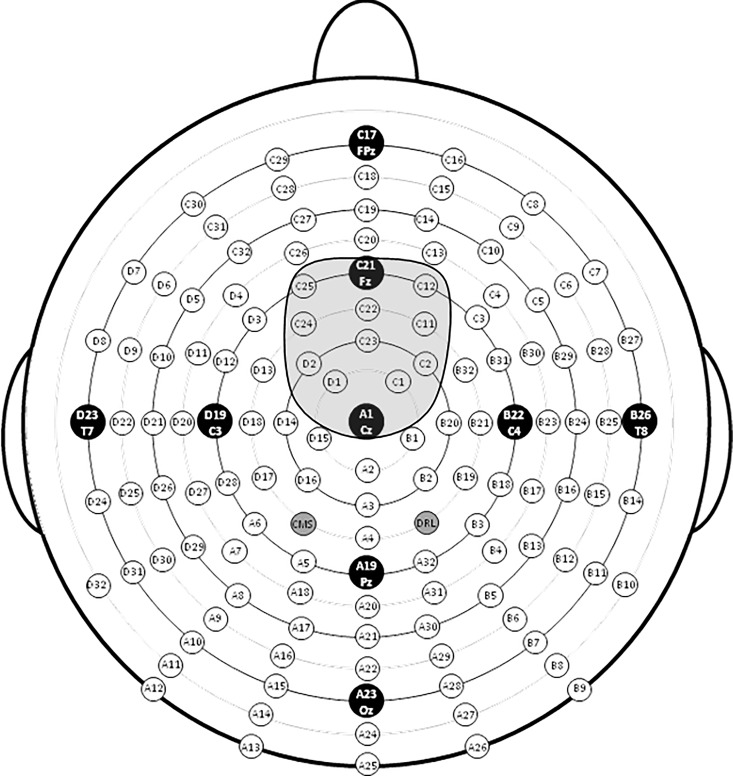
Illustration of the frontocentral cluster of electrode sites used for ERP measurements of the N1 component.

The goal of this study was to examine early auditory processing. We measured the N1, a negative deflection occurring ~100 ms after sound onset. The mean local peak latency of the N1 to novel and standard auditory stimuli was measured between 75 and 175 ms. The N1 amplitude was measured as the mean value of the 50 ms window centered on the mean local peak latency at a cluster of 12 electrodes between Cz and Fz (Fig 2).

## Results

### Participants

Table 1 summarizes participant characteristics, including demographic information and neuropsychological test performance for each age group. A total of 114 individuals participated in the study. There were 23 young, 29 middle-aged, 35 young-old, and 27 old-old adults. An additional 6 young, 4 middle-aged, 4 young-old, and 11 old-old participants completed the experiment, but were excluded due to excessively noisy data. The aforementioned two young participants excluded from behavioral analyses due to missing behavioral data were included in ERP analyses. Of note, the excluded old-old participants and the included old-old participants did not differ in terms of age, years of education, MMSE score, or AMNART IQ scores (*p*s > .4). One-way ANOVAs were run for each of the pertinent demographic variables, with age group as the between subject variable. There were no differences between age groups for EC percentile score based on age-appropriate norms, F(3,110) = .97, *p* = .410, years of education, F(3,110) = .82, *p* = .486, or estimated IQ, F(3,110) = 1.35, *p* = .263. Age groups differed on MMSE score, F(3,110) = 3.15, *p* = .028, η^2^ = .08, such that the young participants had slightly higher scores than the middle-aged (*p* < .05) and old-old participants (*p* < .01). Age groups also differed on mean hearing loss, F(3,110) = 32.41, *p* < .001, η^2^ = .47, such that each age group was different from all others and mean hearing loss increased with age (*p*s < .03). Lastly, age groups differed in visual acuity, F(3,110) = 17.46, *p* < .001, η^2^ = .32, such that young and middle-aged participants did not differ from each other (*p* = .696), and had better visual acuity than young-old participants, who in turn had better visual acuity than old-old participants (*p*s < .01). One old-old participant had visual acuity of 20/80; two old-old participants had visual acuity of 20/50; all other participants had visual acuity of 20/40 or better.

**Table 1:** Demographic and Neuropsychological Information (mean(SD)).

Factors	*Young*	*Middle*	*Young-Old*	*Old-Old*	*p *	η^2^
N	23	29	35	27	-	-
Sex (F:M)	(12:11)	(15:14)	(22:13)	(19:8)	.4	-
Age Range in Years	19–30	40–58	60–79	80–91	-	-
Mean Age in Years	22.9 (2.7)	49.1 (6.0)	71.4 (5.4)	84.1 (2.9)	< .001	.99
Years of Education	15.2 (1.7)	16.1 (2.5)	16.0 (3.1)	16.4 (3.5)	0.5	.02
AMNART IQ	117.6 (6.7)	118.4 (7.9)	119.7 (9.3)	121.85 (8.0)	0.3	.04
MMSE score	29.9 (.3)	29.4 (.8)	29.5 (1.0)	29.11 (1.1)	0.03	.08
EC Percentile Score	67.8 (15.7)	66.8 (13.9)	68.8 (16.4)	73.47 (16.3)	0.4	.03

AMNART = American version of the National Adult Reading Test; MMSE = Mini-Mental State Exam; EC = Executive Capacity

### Behavior

The focus of this study was early processing of irrelevant auditory stimuli. Performance measures were calculated for targets in the visual (auditory-ignore) task preceded by standard and novel auditory stimuli, as well as target trials where there was no preceding auditory stimulus. Table 2 summarizes performance on the visual task and mean number of target letters under the high task load condition.

**Table 2:** Visual Attend (Auditory-Ignore) Task Behavior (mean (SD)).

	Low Task Load		High Task Load		
Age Group	Mean RT (ms)	Accuracy	Mean RT (ms)	Accuracy	Mean Number of Visual Targets
**Young**	489 (47)	0.83 (.12)	602 (62)	0.78 (.11)	7.61 (1.3)
**Middle**	562 (52)	0.92 (.05)	670 (52)	0.82 (.14)	7.97 (1.1)
**Young-old**	585 (49)	0.91 (.08)	709 (48)	0.75 (.15)	6.40 (1.2)
**Old-old**	596 (54)	0.86 (.19)	715 (67)	0.73 (.17)	6.07 (1.5)

RT = Reaction Time

As previously described, the number of target letters during the high task load was titrated to participants' individual performance (aiming for 0.8 accuracy). Number of target letters varied across age groups, F(3,106) = 18.17, *p* < .001, η^2^ = .34. Number of visual targets under high task load was greater for young and middle age groups than for young-old and old-old groups (*p*s < .001), but did not differ between young and middle aged groups (*p* = .262), or between young-old and old-old groups (*p* = .178). Number of target letters under high task load also varied across EC groups, F(1,106) = 11.34, *p* = .001, η^2^ = .12, such that high EC participants were shown more unique target letters (mean = 7.4) than average EC participants (mean = 6.5). There was no interaction between age group and EC group (*p* = .515).

RT and accuracy were analyzed via repeated measures ANOVA across two visual task loads (low and high), visual targets with three different preceding auditory stimulus types (standard, novel, and no stimulus), four age groups, and two EC groups. Mean RT varied across age groups, F(3,104) = 25.66, *p* < .001, η^2^ = .43, such that young participants exhibited the shortest responses, followed by middle-aged, followed by young-old, who were not different from old-old participants. Mean RT also varied across task load, F(1,104) = 885.60, *p* < .001, η^2^ = .90, such that participants responded more quickly under low load than under high load. No interaction was found between load and age group. The type of auditory stimulus preceding visual targets was found to affect RT, F(2,104) = 19.35, *p* < .001, η^2^ = .16. RTs to visual targets preceded by standard and novel sounds did not differ (*p* = .951), but both were shorter than RTs to targets preceded by no auditory stimulus (*p*s < .001).

Accuracy differed between age groups, F(3,104) = 3.21, *p* = .026, η^2^ = .09, and between EC groups, F(1,104) = 7.90, *p* = .006, η^2^ = .07. Age group differences reflected greater accuracy for middle-aged participants compared to old-old participants (*p* = .003); all other age groups did not differ from each other (*p*s > .05). The difference between EC groups was due to better performance by the high EC group (.85) compared to the average EC group (.78). There was no interaction between age group and EC group. Accuracy also varied across visual task load, F(1,104) = 89.52, *p* < .001, η^2^ = .46, such that accuracy was greater during the low load task. In addition, accuracy varied across targets preceded by different stimuli, F(2,104) = 3.89, *p* = .024, η^2^ = .04, such that participants were more accurate responding to visual targets preceded by standard auditory stimuli (mean = .82) than to visual targets preceded by novel auditory stimuli (mean = .81) or no-auditory stimuli (mean = .81) (*p*s < .03). Accuracy rates in response to targets preceded by novel and no-auditory stimuli did not differ (*p* = .523). Although the difference between accuracy in response to visual targets preceded by these three auditory stimulus types is reliable, it is important to note that the mean difference between the no-auditory stimulus and the other two types of stimulus trials was relatively small (.01).

Age group differences in accuracy were modified by task load, F(3,104) = 4.19, *p* = .008, η^2^ = .11, driven by differing age-related accuracy changes between low and high task load. Under low task load, middle-aged and young-old participants performed no differently from each other (*p* = .858) and more accurately than young and old-old participants (*p*s < .02), who did not differ from each other (*p* = .870). Under high task load, old-old participants performed worse than middle-aged participants (*p* < .007), and no other age groups differed in accuracy (*p*s > .07). A correlation analysis between age and the difference in accuracy between high and low task load revealed that the difference in accuracy between high and low task loads increased with age, r = .266, *p* = .005.

During the auditory-attend task, accuracy in response to auditory target stimuli differed across age groups, F(3,104) = 6.53, *p* < .001, η^2^ = .16, with old-old participants performing worse than all other age groups (*p*s < .01), who did not reliably differ from one another (*p*s > .1). The high EC group had higher accuracy than the low EC group, F(1,104) = 8.31, *p* = .005, η^2^ = .07. There was no interaction between age group and EC group (*p* = .198). Mean RT to auditory target stimuli also differed across age groups, F(3,104) = 17.10, *p* < .001, η^2^ = .33, due to young participants having shorter RTs than all other age groups (*p*s < .001), who did not reliably differ from one another (*p*s > .3). There was no effect of EC group, and no interaction between age group and EC.

### ERPs—Average Waveforms

A repeated measures ANOVA on local peak latency measurements revealed no differences between age or EC groups (*p*s > .9). Therefore, the overall mean local peak latency (117 ms) was used to determine the 50 ms measurement window for mean amplitude (92–142 ms). Since primary task load did not vary across the two 4-block sections of the auditory-attend task that differed only by the frequency of individual rare visual stimuli presented, ERPs were averaged across these sections. The mean N1 amplitude across all eight blocks of the auditory-attend task was adopted for comparison with the two loads during the auditory-ignore task in order to avoid arbitrarily dividing 4-block sections of the same task under the same load and to incorporate the greatest number of auditory-attend trials.

**Fig 3:**
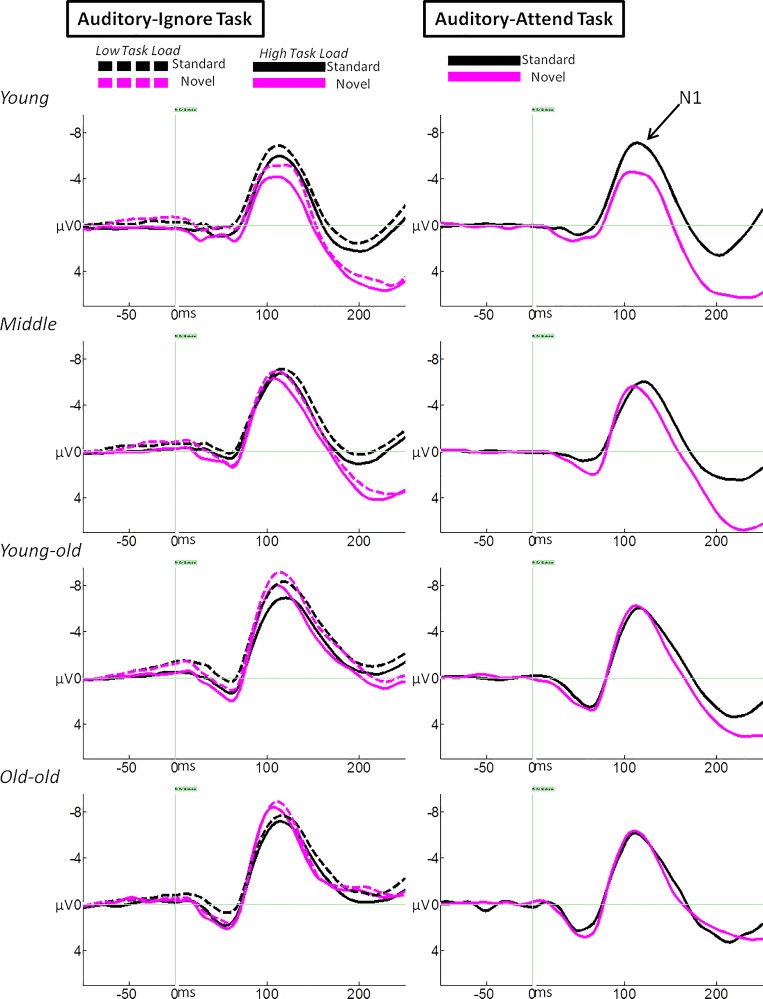
Depiction of the grand average ERP waveforms for all 4 Age Groups. ERPs measured in response to auditory standard and novel stimuli at a cluster of electrodes between Fz and Cz. Arrow indicates the auditory N1 wave.

**Fig 4:**
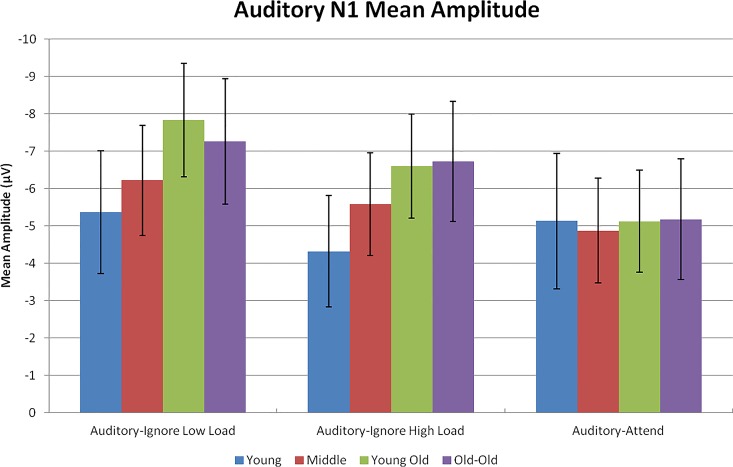
Mean amplitude of the auditory N1. N1 amplitude averaged across standard and novel auditory stimuli for each task and age group. Error bars represent standard deviation.

A repeated measures ANOVA was performed on mean amplitude measurements of the auditory N1 across three tasks (auditory-attend, auditory-ignore low load, and auditory-ignore high load), two auditory stimulus types (standard and novel), two EC groups, and four age groups. Effects and interactions directly related to predictions will be highlighted in this section. The two auditory stimulus types were associated with different patterns of main effects. Those differences, while certainly interesting, are beyond the scope of this paper. The impact of stimulus type will only be discussed if this variable modulates critical findings. Table 3 provides a summary of all effects and interactions. Fig 3 illustrates average waveforms and Fig 4 depicts mean amplitude measurements.

**Table 3:** Average Waveforms Main Effects and Interactions.

ANOVA Main Effects / Interactions	df	F	p	Partial η^2^
Task	2,212	29.42	< .001	0.22
Stimulus Type	1,106	3.16	0.078	0.03
Age Group	3,106	1.65	0.183	0.05
EC Group	1,106	0.36	0.547	0.00
Age Group x EC Group	3,106	0.85	0.472	0.02
Task x Age Group	6,212	4.36	0.001	0.11
Task x EC Group	2,212	0.49	0.587	0.01
Task x Stimulus Type	2,212	3.97	0.024	0.04
Stimulus Type x Age Group	3,106	9.87	< .001	0.22
Stimulus Type x EC Group	1,106	5.31	0.023	0.05
Stimulus Type x Age Group x EC Group	3,106	2.00	0.118	0.05
Task x Age Group x EC Group	6,212	0.72	0.614	0.02
Task x Age Group x Stimulus Type	6,212	0.66	0.663	0.02
Task x Stimulus Type x EC Group	2,212	2.03	0.139	0.02
Task x Stimulus Type x Age Group x EC Group	6,212	0.47	0.808	0.01

**Fig 5:**
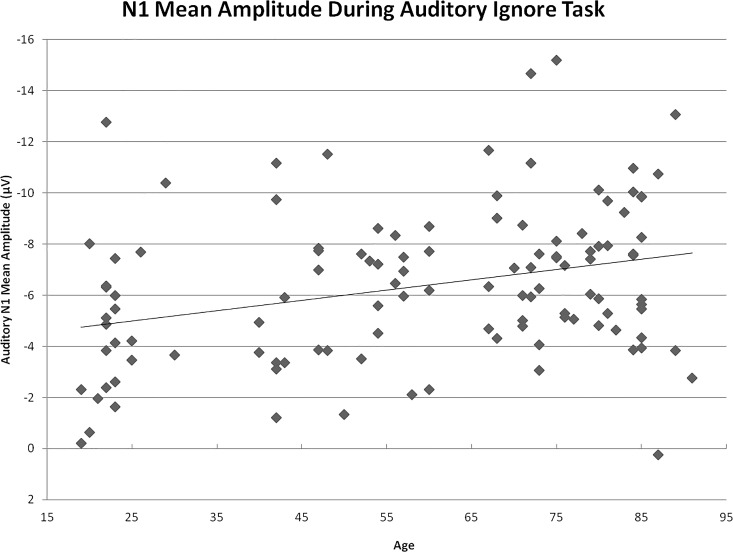
Scatterplot of N1 mean amplitude. Mean amplitude calculated across auditory ignore tasks (low load and high load) and stimulus types. Regression: N1 amplitude (μV) = -3.987–.040 (age).

The interaction between age group and task (*p* = .001) indicated an age-related increase in N1 amplitude to auditory stimuli during the auditory-ignore low load task, F(3,106) = 3.09 *p* = .030, η^2^ = .080, and the auditory-ignore high load task, F(3,106) = 3.18 *p* = .027, η^2^ = .082, but not during the auditory-attend task, F(3,106) = .07, *p* = .974. Regression analysis revealed that the N1 amplitude (collapsed across both loads of the auditory-ignore task and both stimulus types) increased linearly as a function of increasing age, F(1,112) = 11.20, p = .001, R^2^ = .091. For every advancing year in age, the N1 amplitude of participants became larger by -.04 μV (Fig 5). Age was significantly correlated with N1 amplitude across the auditory-ignore tasks, r = -.302, *p* = .001, mean hearing threshold, r = .763, *p* < .001, and visual acuity, r = -.562, *p* < .001. Notably, the correlation between age and N1 amplitude across auditory-ignore tasks remained significant after controlling for mean hearing threshold, r = -.267, *p* = .004, and after controlling for visual acuity, r = -.282, *p* = .002. Age was not associated with N1 amplitude on the auditory-attend task, r = -.032, *p* = .733.

The interaction between age group and task can also be characterized by an effect of task for middle-aged, young-old, and old-old participants, such that the amplitude of the N1 to auditory stimuli was greater during the auditory-ignore tasks than during the auditory-attend task (*p*s < .01), a pattern that was not observed for young adults (*p*s > .07). Of note, the magnitude of the age-related increase in N1 under ignore was not influenced by stimulus type (age group x task x stimulus type interaction, *p* = .663). There was no effect of EC group (*p* = .547), and EC group did not modify any age-related effects (*p*s > .1). There was an effect of task due to differences in N1 amplitude between the auditory-attend, auditory-ignore low load, and auditory-ignore high load tasks (*p*s < .01). In contrast to expectation, N1 amplitude during both auditory-ignore tasks was greater than during auditory-attend task (*p*s < .01). Additionally, N1 amplitude in auditory-ignore low load was larger than during auditory-ignore high load (*p* < .001). To investigate how this difference between auditory-ignore task loads was affected by the interaction with age group, a two task (auditory-ignore low load and auditory-ignore high load) by two stimulus type (standard and novel) by two EC group by four age group ANOVA was performed on auditory N1 amplitude. In the two-task ANOVA, there was an effect of task, F(1,106) = 27.77, *p* < .001, η^2^ = .21, such that N1 amplitude was greater during auditory-ignore low load than during auditory-ignore high load. This effect of task was not modulated by age group, F(3,106) = .72, *p* = .542.

When analyzing overall performance across auditory-ignore low load and auditory-ignore high load tasks, N1 amplitude in response to auditory standard and novel stimuli was not associated with primary (visual) task accuracy, r = -.094, *p* = .324, or primary (visual) task RT, r = -.006, *p* = .954. The inhibitory deficit hypothesis leads to the expectation that older adults who generate the largest N1 amplitude in response to task-irrelevant sounds would perform the worst on the primary visual task. To test this prediction, we repeated the above correlation analyses between visual task performance and N1 amplitude in response to auditory stimuli limited to participants in the middle-aged, young-old, and old-old age groups (n = 91). Among these older participants, a similar set of relationships was revealed: N1 amplitude in response to auditory standard and novel stimuli was not associated with primary (visual) task accuracy, r = -.096, *p* = .366, or primary (visual) task RT, r = .165, *p* = .118. See S1 Text for an analysis of participants matched for EC across age groups using non-age-adjusted scores. The pattern of age-related differences in N1 amplitude in this subsample of participants was similar to that of the entire group.

During the auditory-attend task, N1 amplitude in response to standard stimuli was associated with target RT, r = .292, *p* = .002, and target accuracy, r = -.324, *p* < .001, such that larger N1 amplitudes were linked to better performance (smaller RTs and higher accuracy scores). N1 amplitude in response to novel stimuli was not associated with target RT, r = .058, *p* = .544, or target accuracy, r = -.094, *p* = .323. N1 amplitude in response to either standard or novel stimuli during auditory-attend task was not associated with age (*p*s > .1). See S1 Text, S1 and S2 Tables, and S1 and S2 Figs for an analysis of temporospatial factors representing subcomponents of the N1 derived from PCA. These PCA factors demonstrated effects of task and age group, as well as task x age group interactions similar to those found with the average wave forms.

## Discussion

The primary aim of the current study was to test predictions derived from the inhibitory deficit hypothesis of aging under experimental conditions that controlled for potentially confounding variables. The first prediction, that there would be an age-related increase in early perceptual processing of to-be-ignored auditory stimuli, was confirmed: older adults generated a larger N1 component to task-irrelevant stimuli in the auditory-ignore tasks. The inclusion of an auditory-attend condition that presented identical kinds of physical stimuli, but failed to elicit age-associated increases in the N1 to auditory events, establishes the specificity of enhanced early neural processing of stimuli that are supposed to be ignored. The lack of an age-associated increase in N1 amplitude under the auditory-attend task also strongly argues against the possibility that the age-related findings for the auditory-ignore task were due to enhancing the sound pressure level of stimuli in older adults with documented hearing loss [2].

Results from other recent investigations are consistent with the current study’s pattern of findings. Research using middle latency auditory evoked potentials (6–19 ms after sound onset) [20, 62] and brainstem auditory evoked potentials [63] has shown age-related increases in response to task-irrelevant, to-be-ignored sounds, implicating the involvement of even earlier processing stages. Additionally, the neuromagnetic P1m has been shown to increase with age in response to sounds in a passive listening task [33, 64, 65]. These findings indicate that age-related increases in early processing of unattended auditory stimuli are not limited to a specific component or paradigm. Other studies have demonstrated that under attend conditions, there is no age-related augmentation in the amplitude of ERP markers of early processing of novel sounds [66, 67].

The current investigation succeeded in confirming the major findings of Alain and Woods [2] using a much larger sample of participants that extended the age range to include old-old adults into their 10^th^ decade. Regression analysis suggested a linear relationship between age and the N1 amplitude elicited by task-irrelevant auditory stimuli. In addition, the current study demonstrated that the age-associated increase in the N1 to task-irrelevant auditory stimuli was present even after matching age groups for EC and difficulty of the primary task, as well as after experimentally and statistically controlling for individual hearing threshold. This result strengthens the claim that the differences across groups in the N1 wave were a reflection of aging and not other potential explanatory factors. Moreover, the pattern of age-related response was similar for grand average waveforms and the PCA factors corresponding to the N1, allowing us to more firmly conclude that the results are specific to operations indexed by this component and are not driven by changes in temporally or spatially overlapping components.

Age-associated changes in auditory adaptation may contribute to our findings. Auditory adaptation is defined as the reduction of neural responses to repetitive stimuli over a short time span [68, 69]. One hypothesis is that auditory adaptation reflects an active filtering process that reduces the sensitivity of the auditory system to repeated sounds with limited salience, and enhances sensitivity to novel, more information-rich sounds [70–72]. There is evidence that older adults exhibit diminished auditory adaptation. In response to repetitive auditory stimuli, older individuals demonstrate an attenuation of the N1 component [71, 72] and reduced fMRI activity in auditory and prefrontal cortex [70, 73].

It follows that one source of the age-related increase of the N1 under the auditory-ignore tasks in our experiment may be the diminished tendency of older adults to reduce their N1 response to repetitive standard auditory stimuli. There are several challenges to this hypothesized mechanism. First, the magnitude of the age-related increase in the N1 did not differ in response to repetitive standard stimuli and rare novel stimuli for both the average waveforms and the two PCA factors. Of note, in contrast to standard stimuli, novel auditory stimuli were not repeated over a short temporal interval and the same novel sound stimulus was never presented more than one time. Second, the repetitive standard auditory stimuli were physically identical under the auditory-ignore tasks and the auditory attend task. However, no age-associated increase in N1 was observed under the auditory attend condition.

The behavioral data confirm that the visual high task load condition was more demanding than the low task load condition, with the latter associated with greater accuracy and shorter RTs. Similarly, the behavioral results suggest that individuals with high EC outperform those with average EC on the experimental tasks. Therefore, the unanticipated ERP findings for task load and EC, discussed below, cannot be attributed to a failure of the experimental paradigm to elicit expected behavioral results.

Our findings do not allow us to draw strong inferences regarding whether there were age-related behavioral differences in the distracting effects of task-irrelevant auditory stimuli. The no-auditory stimulus trials prior to the visual events were included to allow for a comparison of participants’ accuracy and RT on such trials with those preceded by auditory events. We did not anticipate finding lower accuracy and prolonged RTs when visual targets were preceded by no-auditory stimuli than by repetitive standard stimuli. In the context of the current investigation, no-auditory stimulus trials were not equivalent to performing the visual task with no auditory distracters. Indeed, the response to targets that revealed the greatest level of distraction in behavioral analyses were those preceded by no-auditory stimuli. These unexpected findings have at least two potential explanations: 1) the standard auditory stimuli may have served as an alerting mechanism prior to a visual event, facilitating a behavioral response, which did not occur on no-auditory stimulus trials [74], or 2) no-auditory stimulus events were infrequent occurrences that may have violated the expectations of participants and thus drawn more attention than repetitive standard stimuli, leading to a disruption of behavioral output. Future studies should compare blocks with and without auditory distracters. If no age-associated increases in the impact of interference were found, it would suggest that during the interval between the augmented early perceptual processing of auditory distracters, indexed by the N1 (~100–150 ms), and the behavioral response, indexed by the button press (~610–630 ms), older adults are able to carry out operations that prevent excessive early processing from causing greater disruption of behavior.

The second predicted outcome derived from the inhibitory deficit hypothesis was that EC would modulate age-related increases in N1 amplitude to auditory stimuli in the visual task. Inhibition is considered one of the critical mechanisms by which executive control is carried out [23, 24, 75]. Consistent with this framework, one would expect that older individuals with higher EC would be more adept at suppressing processing of task-irrelevant auditory stimuli than their peers with lower EC [36, 37]. This prediction was not confirmed. The results of the current cross-modal study are similar to the age-related reduction in inhibitory activity during early selective visual attention to color (in a unimodal task) that has been found even after carefully matching age groups for EC using age-adjusted norms [22]. Taken together, these findings raise questions about the underlying mechanisms that account for the apparent inhibitory deficits observed in cognitive aging. One possibility, which should be investigated by future research, is a dissociation between inhibitory capacity and other executive functions [53, 76].

The third prediction was that the magnitude of age-related increases in early processing of task-irrelevant auditory stimuli, as indexed by N1 amplitude, would be larger under the high load than low load condition. If, as hypothesized, older adults have difficulty maintaining stimulus processing priorities in the face of distracters, one would expect that increasing cognitive load of the primary visual task would deplete limited available resources and lead to greater interference under the high load condition. In fact, the load effect was the opposite of expectation: the N1 amplitude to task-irrelevant auditory events was smaller under the high load condition, a result not influenced by age. One way to account for this unanticipated finding is to suggest that resources not employed in executing the primary visual task may be automatically used to process auditory stimuli without regard for task relevance. Under the low load cognitive task, more resources are available, resulting in increased processing of the goal-irrelevant auditory events. This finding is reminiscent of what Lavie and colleagues have suggested for perceptual, not cognitive, load [1, 25]. Although Perceptual Load Theory has been refined [77, 78] and challenged over time [79], one of its basic tenets is that increased perceptual load of the primary task more fully utilizes capacity in the processing of relevant stimuli. This allows for limited additional capacity for the perception of task-irrelevant stimuli, resulting in diminished processing of events unrelated to task goals [1, 25]. Most of the work in support of this theory has involved unimodal, not cross-modal, paradigms [80]. Our findings raise the possibility that in cross-modal tasks, increased cognitive load of the primary visual task may influence distracter processing in a manner that is similar to increased perceptual load in unimodal tasks. An alternative hypothesis is that the high load condition may elicit stronger attentional engagement in the primary task, marshalling greater recruitment of top-down control activity that limits the processing of irrelevant stimuli in the unattended channel [81–85]. Additional research is necessary to test these competing hypotheses.

Interestingly, the differential impact of unimodal versus cross-modal tests of age-related changes in selective attention has been highlighted by Guerreiro and colleagues [86, 87]. They have contested the universality of the inhibitory deficit hypothesis, arguing that it does not apply to cross-modal paradigms, especially those in which auditory stimuli are presented in the to-be-ignored channel. They cite behavioral results from cross-modal auditory distraction paradigms like the Simon or irrelevant speech tasks, wherein consistent age-related deficits have not been found [87]. Guerreiro and colleagues [87] account for these results by suggesting that distracters presented in the auditory modality can be filtered at both central and peripheral levels of the nervous system. They note that with cross-modal selective attention to the visual modality, “the entire auditory modality can simply be shut off at an early stage” (see Summary and Future Directions in [87]). This mechanism allows for “age-related equivalence…whereby top-down modulation has a larger range of possibilities to suppress the distracting effects of irrelevant auditory information” (see Summary and Future Directions in [87]). The results of our study, which demonstrate a clear age-related increase in the N1 in response to irrelevant auditory information, strongly challenges this hypothesized mechanism. However, our findings raise important questions about ways in which processing cross-modal distracters differ from that of unimodal distracters.

The most unexpected finding of this study is that older adults did not simply fail to suppress the N1 amplitude to auditory stimuli in the task-irrelevant modality; they generated a larger response to task-irrelevant auditory stimuli than to physically identical stimuli in the task-relevant modality. This result is not predicted or explained by the inhibitory deficit hypothesis of cognitive aging, and requires an alternative account.

One suggestion may be derived from other findings of our lab, which have indicated that age-related changes in neural activity are not limited to salient events or task-relevant channels [51, 88–90]. For example, we have observed that age-related processing differences are often as large in response to repetitive standard stimuli as they are to target or novel stimuli [51, 88, 91, 92]. We have hypothesized that older individuals may handle task requirements by adopting a different overall processing strategy or cognitive set [35, 51, 93] from their younger counterparts, which is applied to both salient and non-salient stimulus types. This approach by older adults may reflect the long-term adaptation [93] of an information processing system that, due to the frequent delivery of delayed or degraded perceptual information, is often required to compensate for greater ambiguity regarding the nature of an event [89, 94–97].

For older adults, uncertainty may be managed by augmenting the processing of what would otherwise be considered and processed as non-salient events. Even frequent, repetitive stimuli in a modality that, theoretically, should be ignored, and which young adults would designate as having limited information value, may elicit uncertainty in older adults. Within this framework, the increased early sensitivity to task-irrelevant stimuli in older adults may be an adaptive response to age-associated slowing of processing and motor speed, allowing more time to assess for potential danger and prepare an appropriate behavioral response. Augmented early processing of potential distracters could reflect a kind of 'better-safe-than-sorry' approach, the major disadvantage of which would be taxing the limited resources of the system.

Our hypothesis fails to account for the apparent lack of compensatory activity (i.e., increased N1 amplitude) in the auditory-attend task. Under this condition where auditory stimuli are task-relevant and an overt response is required, it might also be advantageous to generate a larger early response to help counter slowed processing speed and RT, and manage greater uncertainty about the nature of a stimulus. Indeed, this relationship was observed between N1 amplitude in response to repetitive stimuli and performance during the auditory-attend task, wherein a larger N1 response to repetitive stimuli predicted better performance. However, no association between auditory-attend N1 amplitude and age was found. One possible explanation for the dissociation between the type of age-related compensatory activity used to process attended vs. unattended stimuli comes from our prior work on the ERP responses to attended stimuli in the visual modality. We have found that compensatory activity to attended stimuli, as measured by increased ERP amplitude, primarily manifests during late, controlled processing stages, as indexed by the amplitude of the P3, but not during early sensory-perceptual processing, as indexed by the P1 or N1 component [51, 98].

Data from the current study raise the possibility that older adults deal with auditory events occurring outside the focus of attention (in the to-be-ignored modality) using a different strategy, which is linked to the amplification of early stimulus processing. Such an approach might provide a greater opportunity to determine if an unattended auditory event is potentially dangerous and requires the mobilization of a behavioral response. This explanation raises the possibility that the age-associated increase in the N1 component may not reflect a processing failure among older adults (as suggested by the inhibitory deficit hypothesis), but rather an adaptive response. If the inhibitory deficit hypothesis were correct, one might expect that older adults with the largest N1 amplitude to task-irrelevant auditory distracters would have the most impaired performance on the primary visual task [72]. However, no correlation was observed between performance on the primary visual task and N1 amplitude in response to ignored auditory stimuli.

The focus of this study was on age-related differences in the early processing of auditory distracters under conditions that manipulated cognitive load in the visual modality. It was not designed to examine if findings would be similar for visual distracters under conditions manipulating load in the auditory modality. Ideally, future investigations should construct parallel conditions for auditory and visual tasks that would manipulate load in each, and systematically examine the processing of irrelevant stimuli both across and within modalities. Moreover, it would be informative to vary types of stimuli used, the timing of stimulus presentations, and the nature of the primary task demands to determine the extent to which the findings of the current study can be generalized.

## Supporting Information

S1 Fig PCA 101 ms factor: Scalp topographies and waveforms for the four age groups. Responses were averaged across auditory standard and novel stimuli within the auditory-attend condition and across auditory standard and novel stimuli under both task load conditions in the auditory-ignore task.
(TIF)

S2 Fig PCA 144 ms factor: Scalp topographies and waveforms for the four age groups. Responses were averaged across auditory standard and novel stimuli within the auditory-attend condition and across auditory standard and novel stimuli under both task load conditions in the auditory-ignore task.
(TIF)

S1 File Study Data. Raw data analyzed in the current study.
(XLSX)

S1 Table 101 ms factor (TF3SF1) Main Effects and Interactions. (DOCX)

S2 Table 144 ms factor (TF4SF1) Main Effects and Interactions. (DOCX)

S1 Text Additional Waveform and PCA Analyses. Analysis of participants matched for EC across age groups using non-age-adjusted scores and temporospatial factors representing subcomponents of the N1 derived from PCA.
(DOCX)
