# Supplementary figures and images for: Increased Early Processing of Task-Irrelevant Auditory Stimuli in Older Adults
Source: PLoS One. 2016 Nov 2;11(11):e0165645. doi: 10.1371/journal.pone.0165645 (PMC5091907; doi:10.1371/journal.pone.0165645)

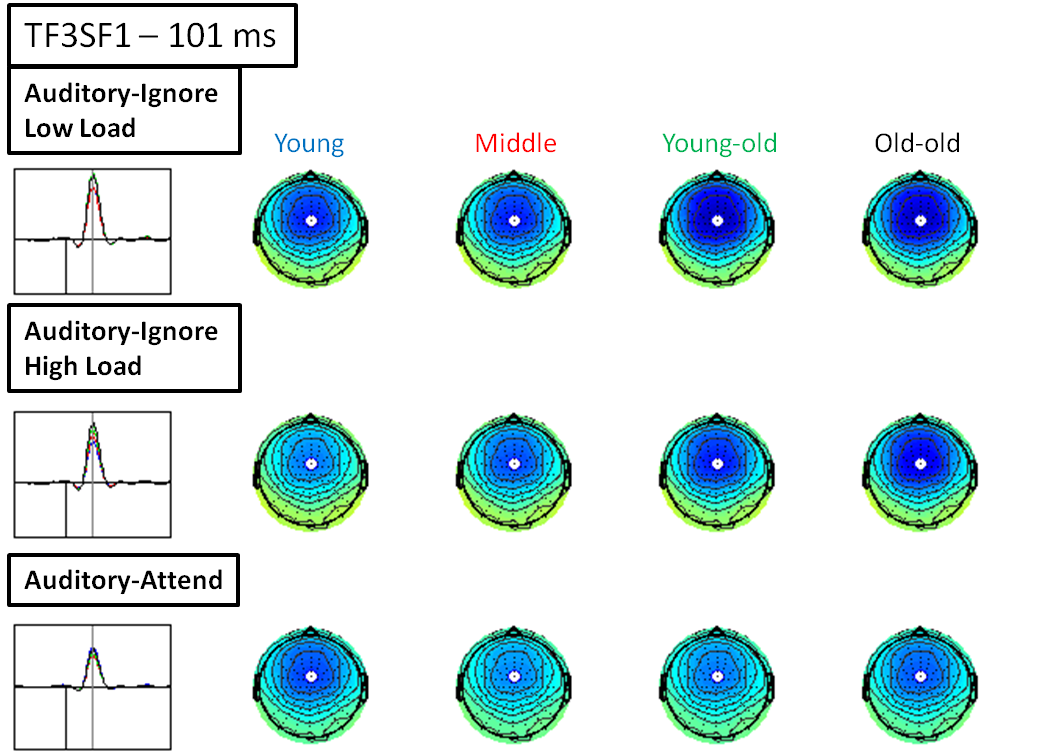

Supplement: S1 Fig — Responses were averaged across auditory standard and novel stimuli within the auditory-attend condition and across auditory standard and novel stimuli under both task load conditions in the auditory-ignore task. (TIF) [file pone.0165645.s001.tif]

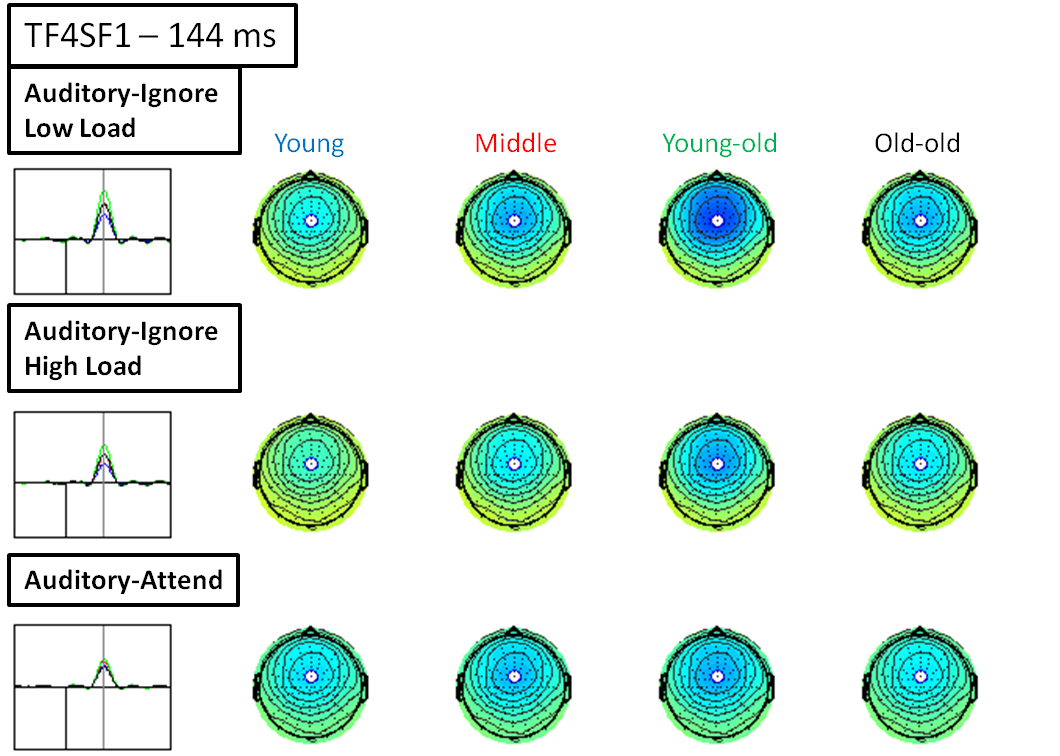

Supplement: S2 Fig — Responses were averaged across auditory standard and novel stimuli within the auditory-attend condition and across auditory standard and novel stimuli under both task load conditions in the auditory-ignore task. (TIF) [file pone.0165645.s002.tif]
