# Supplementary material for: Increased Early Processing of Task-Irrelevant Auditory Stimuli in Older Adults
Source: PLoS One. 2016 Nov 2;11(11):e0165645. doi: 10.1371/journal.pone.0165645 (PMC5091907; doi:10.1371/journal.pone.0165645)
Supplement: S1 Table — (DOCX) [file pone.0165645.s004.docx]

**Supporting Table 1. 101 ms factor (TF3SF1) Main Effects and Interactions**

| **ANOVA Main Effects / Interactions** | **df** | **F** | **p** | **Partial η^2^** |
| --- | --- | --- | --- | --- |
| Task | 2,212 | 28.70 | <.001 | 0.21 |
| Stimulus Type | 1,106 | 0.77 | 0.383 | 0.01 |
| Age Group | 3,106 | 2.84 | 0.041 | 0.07 |
| EC Group | 1,103 | 0.01 | 0.903 | 0.00 |
| Age Group x EC Group | 3,106 | 1.18 | 0.321 | 0.03 |
| Task x Age Group | 6,212 | 6.82 | <.001 | 0.16 |
| Task x EC Group | 2,212 | 1.89 | 0.161 | 0.02 |
| Task x Stimulus Type | 2,212 | 3.81 | 0.025 | 0.03 |
| Stimulus Type x Age Group | 3,106 | 12.61 | <.001 | 0.26 |
| Stimulus Type x EC Group | 1,106 | 4.17 | 0.044 | 0.04 |
| Stimulus Type x Age Group x EC Group | 3,106 | 0.92 | 0.433 | 0.03 |
| Task x Age Group x EC Group | 6,212 | 0.73 | 0.596 | 0.02 |
| Task x Age Group x Stimulus Type | 6,212 | 1.92 | 0.082 | 0.05 |
| Task x Stimulus Type x EC Group | 2,212 | 1.40 | 0.248 | 0.01 |
| Task x Stimulus Type x Age Group x EC Group | 6,212 | 0.52 | 0.783 | 0.01 |
