# Supplementary material for: Increased Early Processing of Task-Irrelevant Auditory Stimuli in Older Adults
Source: PLoS One. 2016 Nov 2;11(11):e0165645. doi: 10.1371/journal.pone.0165645 (PMC5091907; doi:10.1371/journal.pone.0165645)
Supplement: S2 Table — (DOCX) [file pone.0165645.s005.docx]

**Supporting Table 2. 144 ms factor (TF4SF1) Main Effects and Interactions**

| **ANOVA Main Effects / Interactions** | **df** | **F** | **p** | **Partial η^2^** |
| --- | --- | --- | --- | --- |
| Task | 2,212 | 11.40 | <.001 | 0.10 |
| Stimulus Type | 1,106 | 17.92 | <.001 | 0.14 |
| Age Group | 3,106 | 6.16 | 0.001 | 0.15 |
| EC Group | 1,103 | 0.08 | 0.772 | 0.00 |
| Age Group x EC Group | 3,106 | 1.41 | 0.245 | 0.04 |
| Task x Age Group | 6,212 | 4.68 | 0.001 | 0.12 |
| Task x EC Group | 2,212 | 0.10 | 0.862 | 0.00 |
| Task x Stimulus Type | 2,212 | 1.60 | 0.205 | 0.01 |
| Stimulus Type x Age Group | 3,106 | 2.97 | 0.035 | 0.08 |
| Stimulus Type x EC Group | 1,106 | 0.15 | 0.701 | 0.00 |
| Stimulus Type x Age Group x EC Group | 3,106 | 0.63 | 0.596 | 0.02 |
| Task x Age Group x EC Group | 6,212 | 0.64 | 0.661 | 0.02 |
| Task x Age Group x Stimulus Type | 6,212 | 1.81 | 0.101 | 0.05 |
| Task x Stimulus Type x EC Group | 2,212 | 2.12 | 0.124 | 0.02 |
| Task x Stimulus Type x Age Group x EC Group | 6,212 | 0.50 | 0.807 | 0.01 |
