# Supplementary material for: Increased Early Processing of Task-Irrelevant Auditory Stimuli in Older Adults
Source: PLoS One. 2016 Nov 2;11(11):e0165645. doi: 10.1371/journal.pone.0165645 (PMC5091907; doi:10.1371/journal.pone.0165645)
Supplement: S1 Text — Analysis of participants matched for EC across age groups using non-age-adjusted scores and temporospatial factors representing subcomponents of the N1 derived from PCA. (DOCX) [file pone.0165645.s006.docx]

**Average Waveform Analysis - Non-Age-Adjusted EC Norms**

Although age groups were matched for EC using age-adjusted norms, the non-adjusted scores of older subjects on tests of executive function were lower than those of their younger counterparts. To address this issue, we created a non-age-adjusted EC matched subsample by comparing young subjects with average EC, middle-aged subjects with average EC, young-old subjects with high EC, and old-old subjects with high EC (after excluding the two lowest performers of the latter group). Non-age-adjusted percentile scores were calculated for the middle-aged, young-old, and old-old subjects using young adult norms, in order to create a consistent measure across different tests and age groups. Within this subsample of subjects, the non-age-adjusted EC percentile score did not differ across age groups, F(3,55) = 1.98, *p* > .1. Under the auditory-ignore task, age predicted N1 amplitude, r = -.33, *p* < .05, such that older subjects displayed larger N1 amplitude. Under the auditory-attend task, there was no relationship between age and N1 amplitude, r = -.10 *p* > .5. Also, for this subset of middle-aged, young-old, and old-old subjects, N1 was larger under both auditory-ignore loads than during auditory-attend (*p*s < .05), whereas for young adults there was no difference in N1 across tasks (*p* > .7). Thus, the pattern of age-related differences in N1 amplitude to auditory stimuli in this subsample of subjects with matched EC percentile scores using non-age-adjusted norms was similar to that of the entire group of subjects wherein EC percentile scores were calculated using age-appropriate norms.

**PCA – Methods**

Following the recommendation of Dien [1] and our own past work [2, 3], a temporospatial PCA (temporal PCA followed by spatial PCA) was conducted on averaged trials for each individual participants at all 134 electrode sites. ERPs to auditory standard, novel, and rare stimuli, under the auditory-attend and auditory-ignore task conditions for both low and high visual task load, were included in the analysis. Although the results for auditory rare (target) stimuli are not presented, these stimuli were included in the PCA in order to increase variance upon which this analysis depends [1].

Utilizing the ERP PCA toolkit 2.38 [4], a Promax rotation was used and a covariance matrix and Kaiser normalization were applied to the data. Each dataset consisted of 153 time points between -200 and 400 ms. A parallel test was used to restrict the number of factors generated for each PCA. Consistent with the literature, factors of interest were selected based on visual inspection of the timing and topography of the output [5-8]. Any factors that accounted for > 2% of the total variance were considered for further analyses [1].

**PCA - Results**

Two temporospatial PCA factors were identified as subcomponents of the N1 wave based on their frontocentral spatial topographies and peak latencies within the range of the N1: spatial factor 1 in temporal factor 3 (TF3SF1), peaking at 101 ms (101 ms factor), and spatial factor 1 in temporal factor 4 (TF4SF1), peaking at 144 ms (144 ms factor). The 101 ms factor accounted for 7.6% of total variance, and the 144 ms factor accounted for 5.1%. In line with the average waveform analysis, ERPs were averaged across the two 4-block sections of the auditory-attend task that only varied in terms of the frequency of the rare, task-irrelevant visual letters. A repeated measures ANOVA was performed on PCA factor scores at 101 ms and 144 ms across three tasks (auditory-attend, auditory-ignore low load, and auditory-ignore high load), two auditory stimulus types (standard and novel), two EC groups, and four age groups. See Tables S1 and S2 for a summary of all effects and interactions in the 101 ms and 144 ms factors, and Figs S3 and S4 for waveforms and topographic maps.

There were main effects of task and age group as well as task x age group interactions for both the 101 ms and 144 ms PCA factors. The statistical patterns found for the N1 PCA factors were consistent with those observed for N1 mean amplitude derived from average waveforms. For both the 101 ms and 144 ms factors, an age-related increase in factor amplitude was observed for both the auditory-ignore low load and auditory-ignore high load tasks (*p*s < .01), but not for the auditory-attend task (*p* > .3). Additionally, for the 101 ms factor, middle aged, young-old, and old-old age groups displayed a larger factor amplitude during both auditory-ignore tasks than during the auditory-attend task (*p*s < .01), while the young age group did not show reliable differences (*p*s > .1). For the 144 ms factor, a larger factor amplitude was observed for young-old and old-old age groups during the low and high load auditory ignore tasks than during the auditory attend task (*p*s < .01), whereas the young age group tended to exhibit a different pattern: greater amplitude during the auditory-attend than during the auditory-ignore high load task (*p* = .006), but no difference between auditory-attend and auditory-ignore low load (*p* = .196). Neither EC group nor stimulus type modulated the task x age group interaction for either factor (*p*s > .08).

**References**

1. Dien J. Applying principal components analysis to event-related potentials: a tutorial. Dev Neuropsychol. 2012;37(6):497-517. doi: 10.1080/87565641.2012.697503. PubMed PMID: 22889342.

2. Alperin BR, Mott KK, Rentz DM, Holcomb PJ, Daffner KR. Investigating the age-related "anterior shift" in the scalp distribution of the P3b component using principal component analysis. Psychophysiology. 2014;51(7):620-33. Epub 2014/03/26. doi: 10.1111/psyp.12206. PubMed PMID: 24660980.

3. Alperin BR, Tusch ES, Mott KK, Holcomb PJ, Daffner KR. Investigating age-related changes in anterior and posterior neural activity throughout the information processing stream. Brain Cogn. 2015;99:118-27. Epub 2015/08/22. doi: 10.1016/j.bandc.2015.08.001. PubMed PMID: 26295684; PubMed Central PMCID: PMC4605281.

4. Dien J. The ERP PCA Toolkit: an open source program for advanced statistical analysis of event-related potential data. J Neurosci Methods. 2010;187(1):138-45. Epub 2009/12/29. doi: 10.1016/j.jneumeth.2009.12.009. PubMed PMID: 20035787.

5. Dien J, Spencer KM, Donchin E. Localization of the event-related potential novelty response as defined by principal components analysis. Brain Res Cogn Brain Res. 2003;17(3):637-50. PubMed PMID: 14561451.

6. Goldstein A, Spencer KM, Donchin E. The influence of stimulus deviance and novelty on the P300 and novelty P3. Psychophysiology. 2002;39(6):781-90. PubMed PMID: 12462506.

7. Spencer KM, Dien J, Donchin E. A componential analysis of the ERP elicited by novel events using a dense electrode array. Psychophysiology. 1999;36:409-14.

8. Spencer KM, Dien J, Donchin E. Spatiotemporal analysis of the late ERP responses to deviant stimuli. Psychophysiology. 2001;38(2):343-58. PubMed PMID: 11347879.
